# Supplementary material for: Integrating taxonomic and phenotypic information through FISH-enhanced flow cytometry for microbial community dynamics analysis
Source: Microbiol Spectr. 2025 Jun 23;13(8):e01973-24. doi: 10.1128/spectrum.01973-24 (PMC12323603; doi:10.1128/spectrum.01973-24)
Supplement: Supplemental materials — Supplemental figures, text, and tables. [file spectrum.01973-24-s0001.docx]

## Supplementary information

### Supporting information Materials and methods: Sampling (S1)

The marine ecosystem samples originated from the effluent of four trickling filter bioreactors, two containing the plastic PHBH (poly(3-hydroxybutyrate-co-3-hydroxyhexanoate)) and two containing a novel plastic, referred to as B4PF01 (PI-protected chemical composition), set up to investigate plastic biodegradation (unpublished data). Effluent samples for immediate fixation and flow cytometry analysis were collected every Monday, Wednesday, and Friday for a total of 56 days. Immediately after sampling, samples were fixed. Samples for 16S rRNA amplicon sequencing were collected on day 0, 14, 28, 35, 42 and 56. Samples (2 mL) were centrifuged at max speed for 10 minutes and the pellet was stored at –20°C until further analysis.

*In vitro* simulated gut microbiome samples were derived from the Simulator of the Human Intestinal Microbial Ecosystem (SHIME, Prodigest, Zwijnaarde, Belgium), a dynamic *in vitro* gut model consisting of a series of connected double-jacketed vessels, simulating specific regions of the gastrointestinal tract, with a controlled pH, residence time, temperature (37°C), mixing (200 rpm) and diet(Minnebo et al., 2021; Molly et al., 1993; Van de Wiele et al., 2015). The set-up comprised three connected vessels representing a combined stomach and small intestine, a proximal and a distal colon. The proximal and distal colon pH of 5.6-5.9, respectively, 6.6-6.9, was maintained with built-in pH controllers and pumps regulating the dosage of 0.5 M NaOH and HCl (Chem Lab, Zedelgem, Belgium). Two different setups were used in the experiment, which each ran in duplicate, resulting in a quadruple setup. The first setup was a standardised SHIME, which operated with standardised eating patterns (three meals provided daily at 09.00, 17.00 and 01.00h), and standardised nutritional media (Prodigest, Zwijnaarde, Belgium) of which 140 mL was provided and mixed with 60 mL pancreatic juice to the proximal colon vessel per meal. With standardised volumes of 500 and 800 mL respectively, this results in standardised residence times of 24 h in the proximal and 32 h in the distal colon. The second setup was an individualised SHIME, in which the transit time, eating patterns and nutritional medium were adjusted according to the faecal donor characteristics, captured in a food frequency questionnaire according to Minnebo et al (unpublished data).

A faecal donor sample was used to inoculate the colon vessels of the SHIME. The system was allowed to stabilise for 11 days until a stable microbial community was established. Samples were collected on days 1, 2, 4, 7, 9 and 11 post-inoculation for analysis (FCM and 16S rRNA amplicon sequencing). These *in vitro* simulated gut microbial samples were immediately fixed.

**Supplementary Table 1** – Summary of the different FISH probes used in this research.

| **Probe** | **Target** | **Sequence (5’ 🡪 3’)** | **rRNA target site (position)** | **Fluorescent tag** | **Reference** |
| --- | --- | --- | --- | --- | --- |
| EUB338 I | domain *Bacteria* (except * and **) | GCT GCC TCC CGT AGG AGT | 16S, 338-355 | Atto490ls | (R. I. Amann et al., 1990) |
| EUB338 II | **Planctomycetales* (69% coverage) | GCA GCC ACC CGT AGG TGT | 16S, 338-355 | Atto490ls | (Daims et al., 1999) |
| EUB 338 III | ** *Verrumicrobiales* | GCT GCC ACC CGT AGG TGT | 16S, 338-355 | Atto490ls | (Daims et al., 1999) |
| GAM42a | *Gammaproteobacteria* | GCC TTC CCA CAT CGT TT | 23S, 1027-1043 | Cy5 | (Manz et al., 1992) |
| cGAM42a-Quencher | Competitor probe *Gamma- proteobacteria* | GCC TTC CCA CTT CGT TT | 23S, 1027-1043 |  | (Manz et al., 1992) |
| ALF968 | *Alphaproteobacteria* | GGT AAG GTT CTG CGC GTT | 16S, 968-985 | FITC | (A, 1997) |
| LGC 354A | *Bacillota* | TGG AAG ATT CCC TAC TGC | 16S, 354-371 | FITC | (Meier et al., 1999) |
| LGC 354B | *Bacillota* | CGG AAG ATT CCC TAC TGC | 16S, 354-371 | FITC | (Meier et al., 1999) |
| LGC 354C | *Bacillota* | CCG AAG ATT CCC TAC TGC | 16S, 354-371 | FITC | (Meier et al., 1999) |
| CFB719 | *Bacteroidetes* | AGC TGC CTT CGC AAT CGG | 16S, 719-736 | Cy5 | (Weller et al., 2000) |

**Supplementary Table 2** – Reducing anaerobic phosphate buffer (pH 6.8) composition. The phosphate buffer was sparged and flushed with N2-gas prior to autoclaving. Reproduced from Minnebo et al. (2021) (Minnebo et al., 2021).

**Component Composition (g L-1) Molar conc. (mM) Manufacturer**

L-Cystein- HCl 1.00 6.34e-3 J&K Scientific bvba, Lommel, Belgium

C_2_H_3_O_2_SNa 1.00 8.75e-3 Sigma Aldrich, St. Louis, MO, USA

NaHCO 31.40 2.40e-2 Chem-lab, Zedelgem, Belgium

NaCl 0.90 1.07e-2 Carl Roth, Karlsruhe, Germany

KH_2_PO_4_ 6.80 5.00e-2 Carl Roth, Karlsruhe, Germany

K_2_HPO_4_ 8.72 5.00e-2 Carl Roth, Karlsruhe, Germany

**Supplementary Table 3** – By means of three different statistical tests, the multivariate data of the marine trickling filter samples was analysed. The Bray-Curtis dissimilarity matrix was used to test the distance between the centroids of the groups with different plastics (F and P); the effect size was calculated and the variation within the groups was calculated and compared (α=0.05; *** p≤0.001; ** p≤0.005; * p≤0.05).

| **Method** | **Test** | **Distance matrix** | **Groups** | **Statistic** | **P-value** |
| --- | --- | --- | --- | --- | --- |
| 16S | PERMANOVA: effect size | Bray | P-F | 43.24 | 0.001*** |
|  | Permutation test for homogeneity of multivariate dispersions | Bray | P-F | 19.29 | 0.001*** |
| Fixed_DAPI | PERMANOVA: distance | Bray | P-F | 0.20 | 0.002** |
|  | PERMANOVA: effect size | Bray | P-F | 9.6941 | 0.001*** |
|  | Permutation test for homogeneity of multivariate dispersions | Bray | P-F | 8.5066 | 0.004** |
| Fixed_SG | PERMANOVA: distance | Bray | P-F | 0.19 | 0.004** |
|  | PERMANOVA: effect size | Bray | P-F | 4.7158 | 0.001*** |
|  | Permutation test for homogeneity of multivariate dispersions | Bray | P-F | 1.3155 | 0.246 |
| Non-fixed_SG | PERMANOVA: distance | Bray | P-F | 0.15 | 0.006** |
|  | PERMANOVA: effect size | Bray | P-F | 3.9842 | 0.005** |
|  | Permutation test for homogeneity of multivariate dispersions | Bray | P-F | 1.6915 | 0.212 |
| Fixed_FISH | PERMANOVA: distance | Bray | P-F | 0.32 | 0.001*** |
|  | PERMANOVA: effect size | Bray | P-F | 17.557 | 0.001*** |
|  | Permutation test for homogeneity of multivariate dispersions | Bray | P-F | 0.9189 | 0.37 |

**Supplementary Table 4** – By means of three different statistical tests, the multivariate data of the simulated gut samples was analysed. The Bray-Curtis dissimilarity matrix was used to test the distance between the centroids of the individualised and standardised group (Individualised SHIME – IS and Standardised SHIME – SS); the effect size was calculated and the variation within the groups was calculated and compared (α=0.05; *** p≤0.001; ** p≤0.005; * p≤0.05).

| **Method** | **Test** | **Distance matrix** | **Groups** | **Statistic** | **P-value** |
| --- | --- | --- | --- | --- | --- |
| 16S | PERMANOVA: effect size | Bray | IS-SS | 10.347 | 0.001*** |
|  | Permutation test for homogeneity of multivariate dispersions | Bray | IS-SS | 3.6177 | 0.077 |
| Fixed PFA | PERMANOVA: distance | Bray | IS-SS | 0.2248 | 0.001*** |
| FISH | PERMANOVA: effect size | Bray | IS-SS | 11.223 | 0.001*** |
|  | Permutation test for homogeneity of multivariate dispersions | Bray | IS-SS | 6.9925 | 0.011* |
| Fixed EtOH | PERMANOVA: distance | Bray | IS-SS | 0.1949 | 0.001*** |
| FISH | PERMANOVA: effect size | Bray | IS-SS | 6.814 | 0.001*** |
|  | Permutation test for homogeneity of multivariate dispersions | Bray | IS-SS | 4.308 | 0.057 |
| Non-fixed_SGPI | PERMANOVA: distance | Bray | IS-SS | 0.2632 | 0.001*** |
|  | PERMANOVA: effect size | Bray | IS-SS | 8.6234 | 0.001*** |
|  | Permutation test for homogeneity of multivariate dispersions | Bray | IS-SS | 0.3067 | 0.609 |


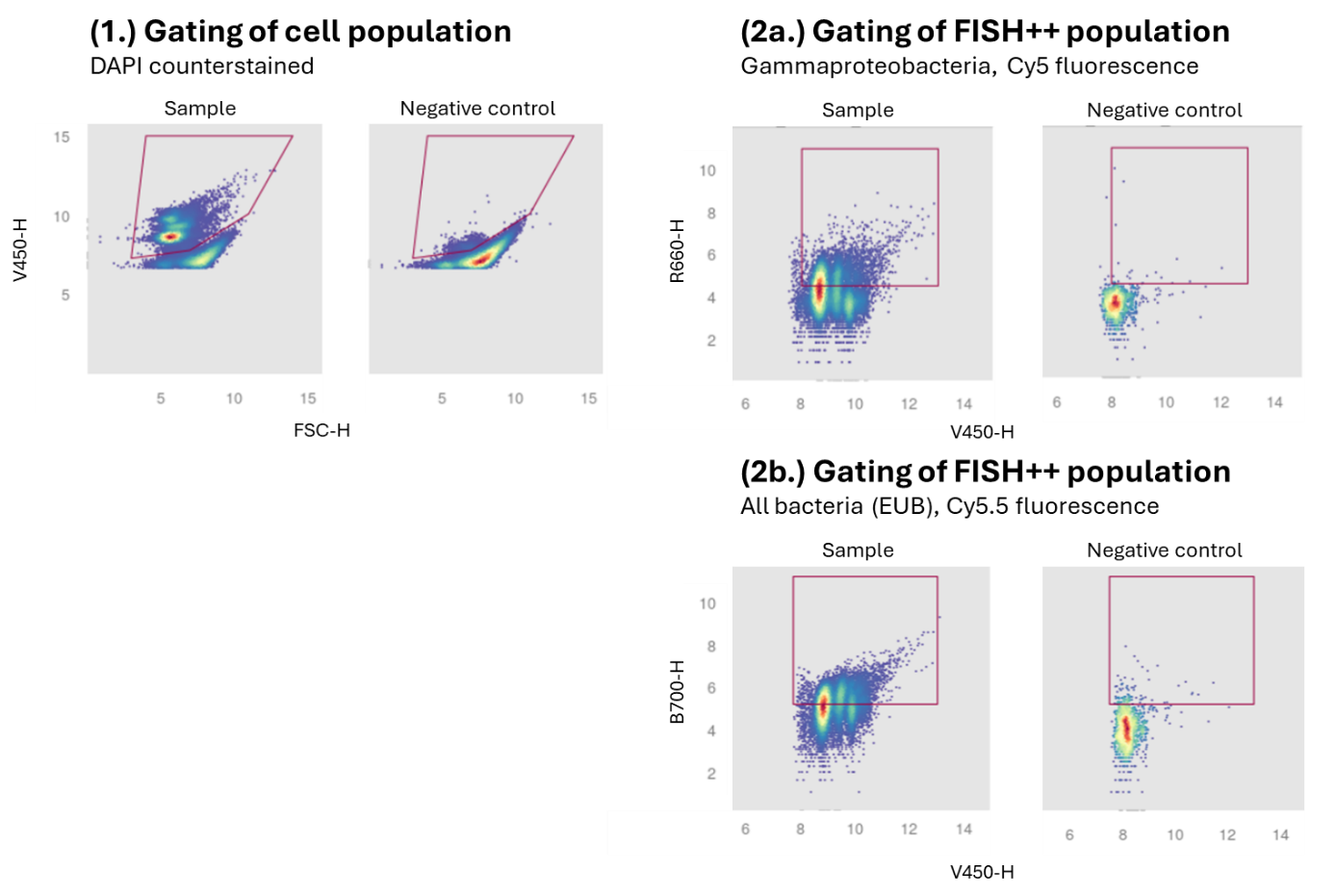
**Supplementary Figure 1** – Illustration of gating strategy, samples originating from the marine trickling filter setup. (1.) Cells are stained by a generic DNA stain and can be separated from the background, measured by a negative control sample. (2.) Cells with high fluorescent signal from FISH labelling can be distinguished from the negative control and from cells without sufficient FISH fluorescence: illustrated for the Gammaproteobacteria probe (2a.) and the general EUB probes mix (2b.).


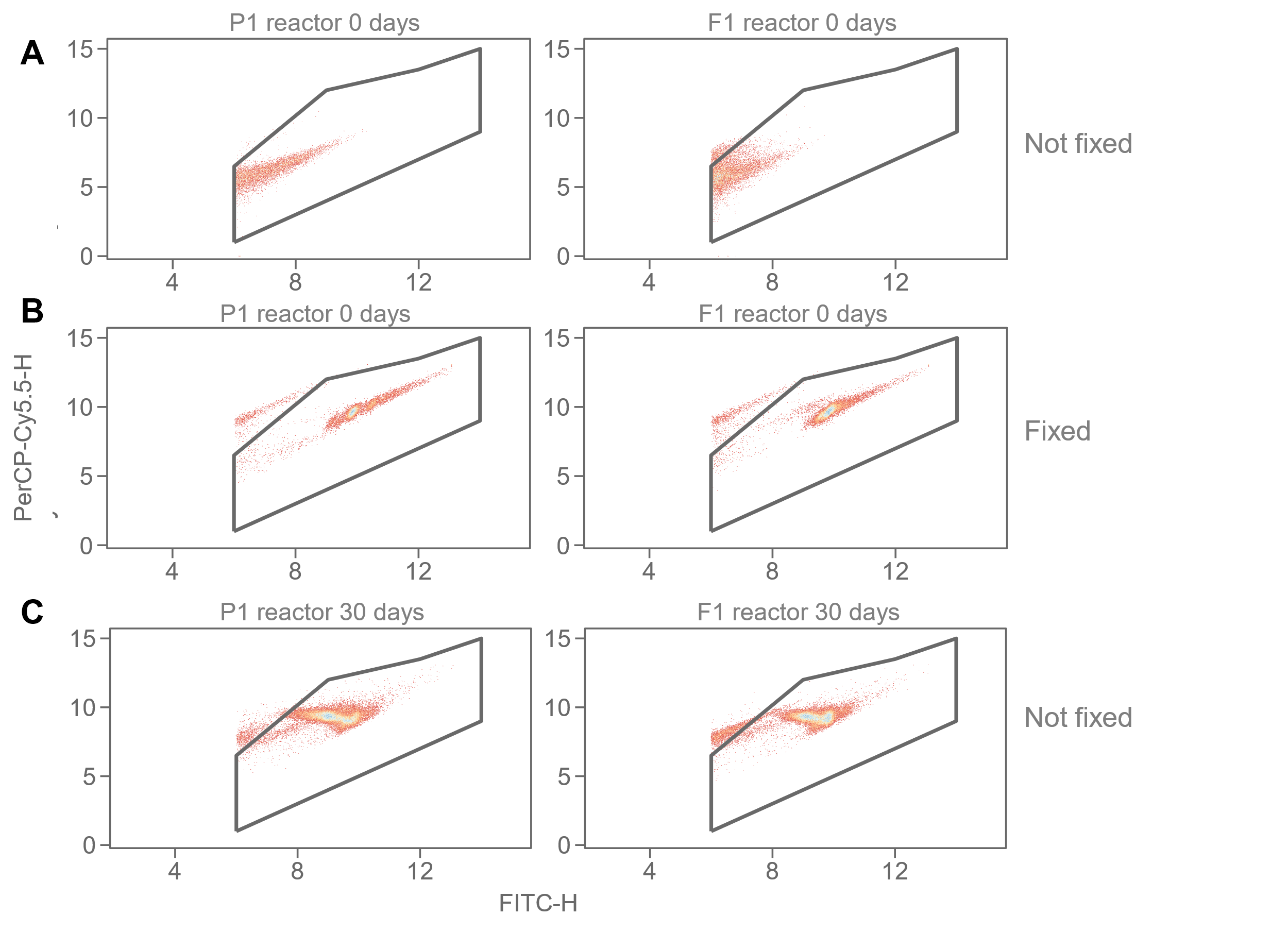
**Supplementary Figure 2** – (**A**) Samples of day 0 of both B4PF01- and PHBH-fed reactors (the marine trickling filter experiment) (replicate P1 and F1), not fixed, stained with SG; (**B**) Samples day 0 of both B4PF01- and PHBH-fed reactors (replicate P1 and F1), fixed, stained with SG and (**C**) Samples from day 30 and 32 of both B4PF01- and PHBH-fed reactors (replicate P1 and F1), not fixed, SG stained.


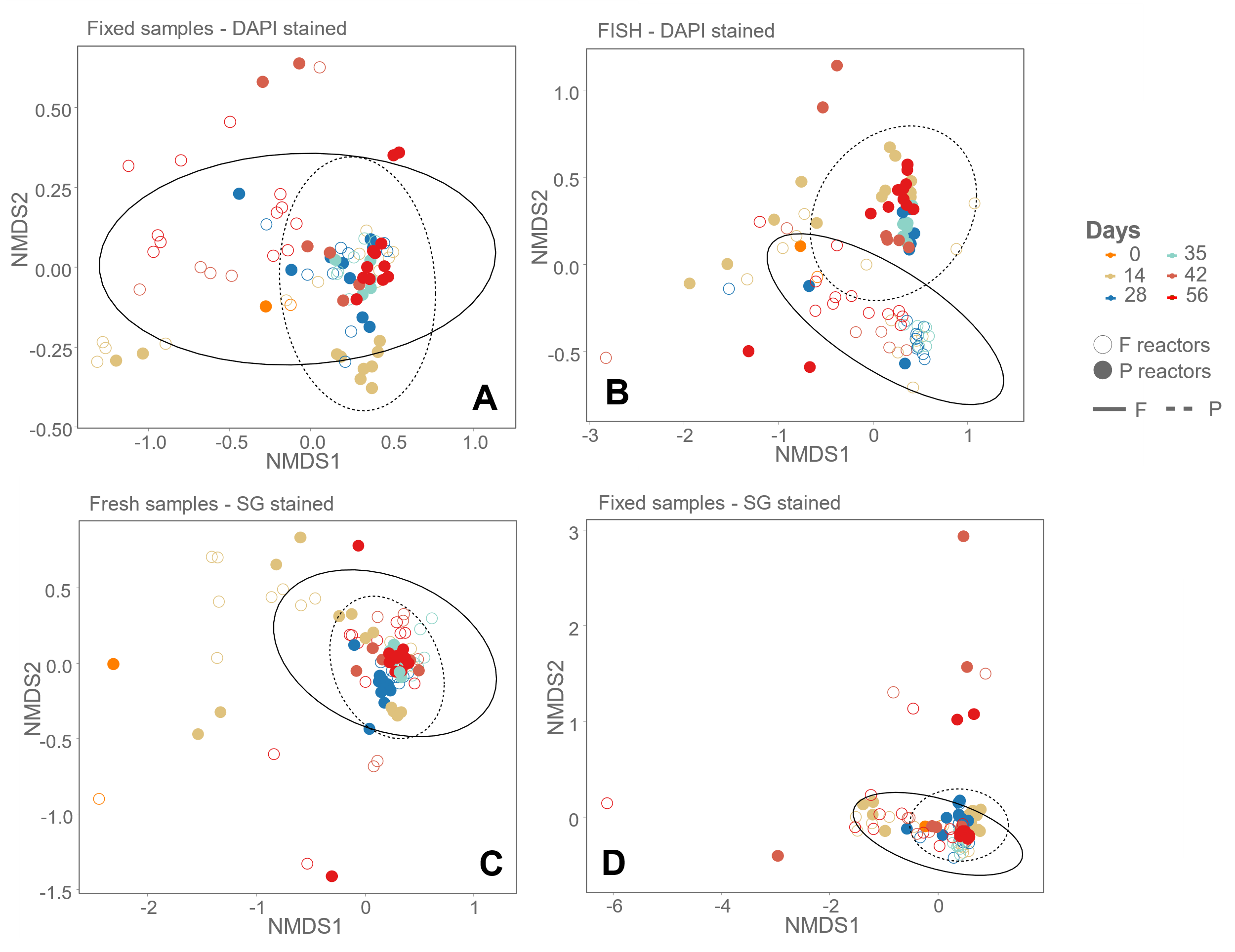


**Supplementary Figure 3** – Marine trickling filter experiment. NMDS ordination based on Bray-Curtis dissimilarity matrix calculated from (**A**) DAPI staining of PFA-fixed cells, (**B**) FISH and DAPI staining of PFA-fixed cells, (**C**) SG staining of live cells, and (**D**) SG staining of PFA-fixed cells. The different reactors, based on plastic material (P: PHBH-fed, F: B4PF01-fed) are displayed by empty (○) and full (●) circles. The ellipse is drawn on the 95% confidence level.


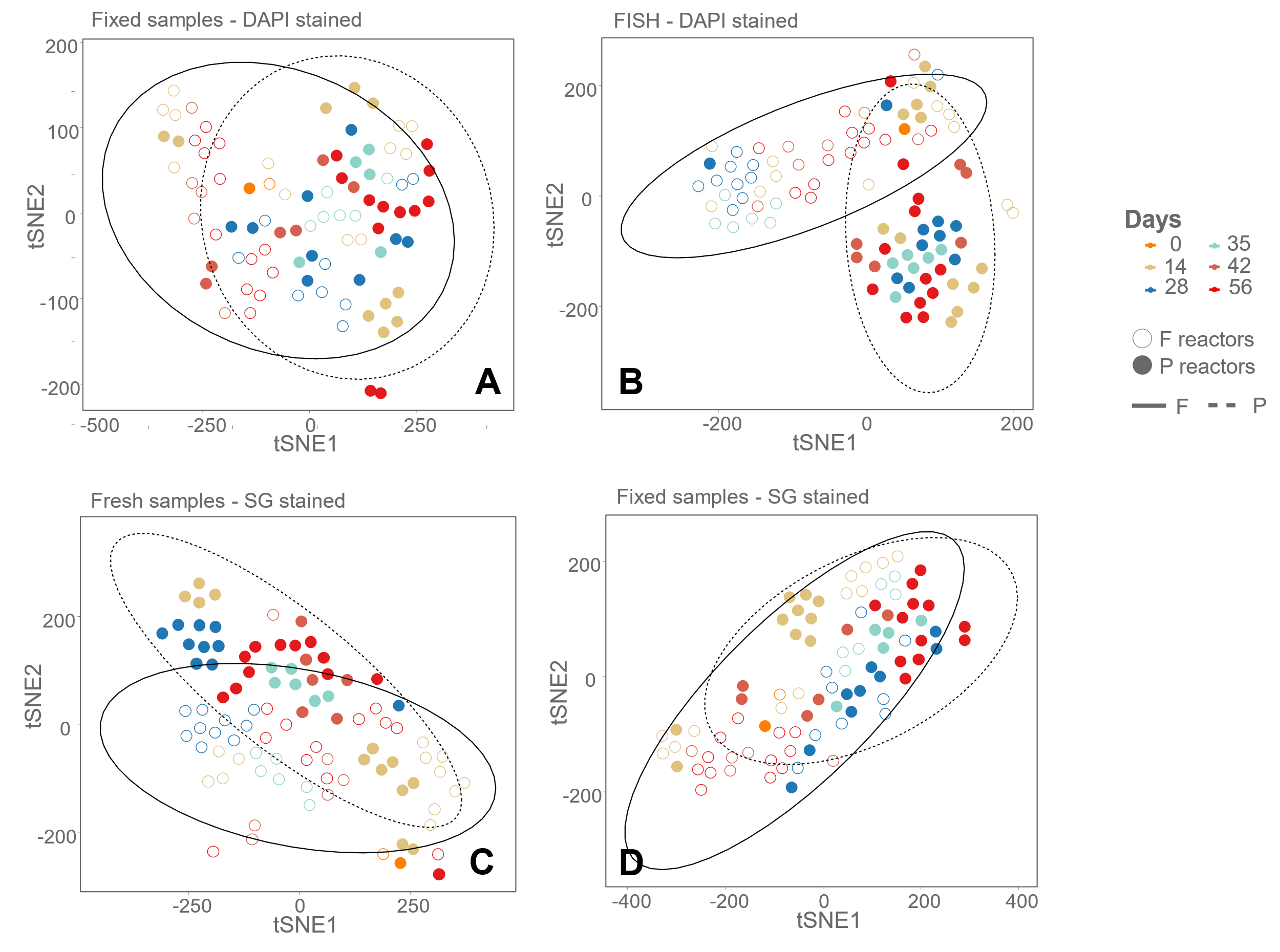


**Supplementary Figure 4** – Marine trickling filter experiment. tSNE dimensionality reduction based on Bray-Curtis dissimilarity matrix calculated from (**A**) DAPI staining of PFA-fixed cells, (**B**) FISH and DAPI staining of PFA-fixed cells, (**C**) SG staining of live cells, and (**D**) SG staining of PFA-fixed cells. The different reactors, based on plastic material (P: PHBH-fed, F: B4PF01-fed) are displayed by empty (○) and full (●) circles. The ellipse is drawn on the 95% confidence level.


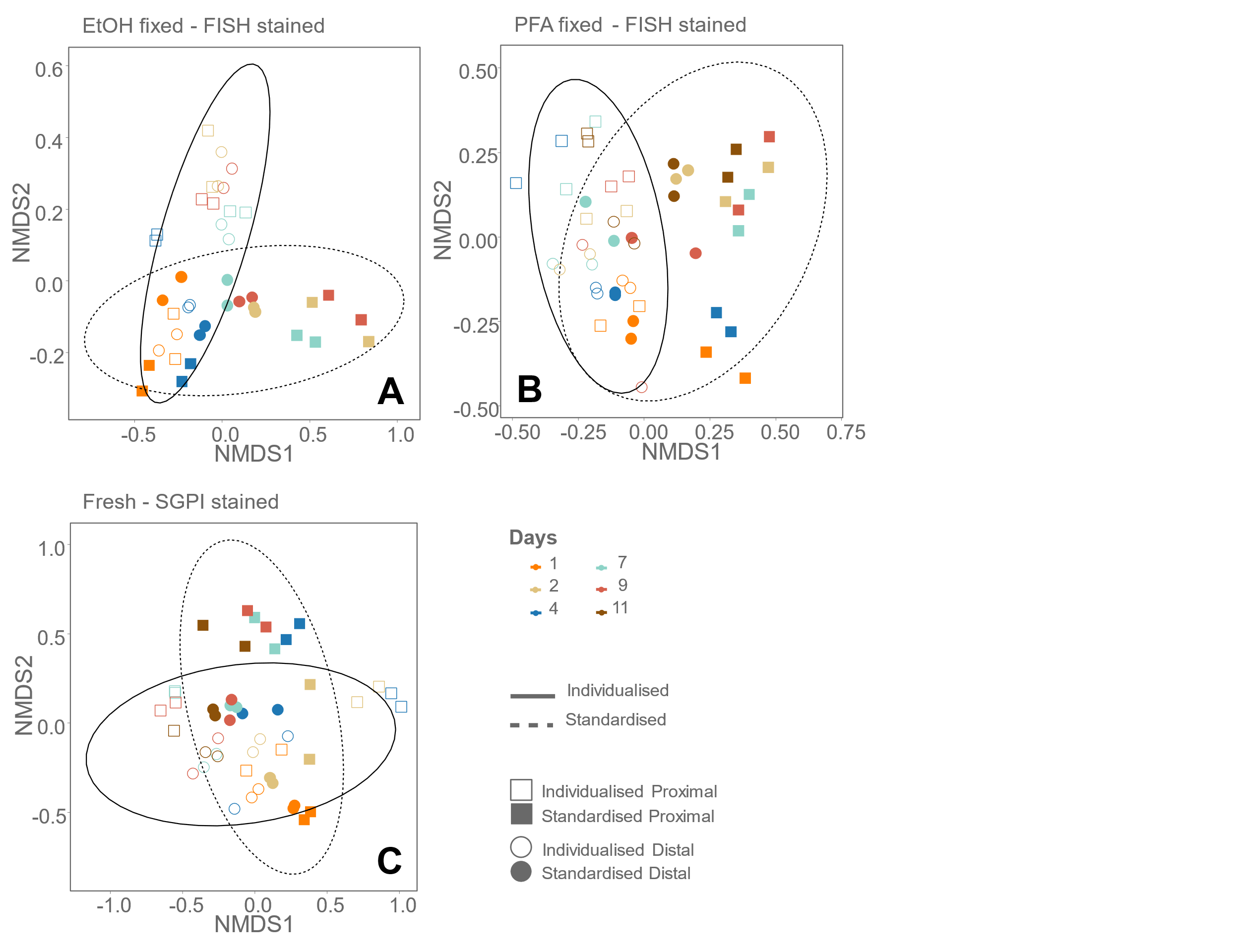


**Supplementary Figure 5** – Simulated gut experiment. NMDS ordination based on Bray-Curtis dissimilarity matrix calculated based on (**A**) DAPI and FISH staining of EtOH -fixed cells (**B**) DAPI and FISH staining of PFA-fixed cells and (**C**) SGPI staining. The different reactors, based on individualised/standardised SHIME operation and distal/proximal colon vessel, are displayed by empty (○,□) and full (●, ■) circles and squares. Biological duplicates are shown. The ellipse is drawn on the 95% confidence level (based only on SHIME operation settings: individualised and standardised).


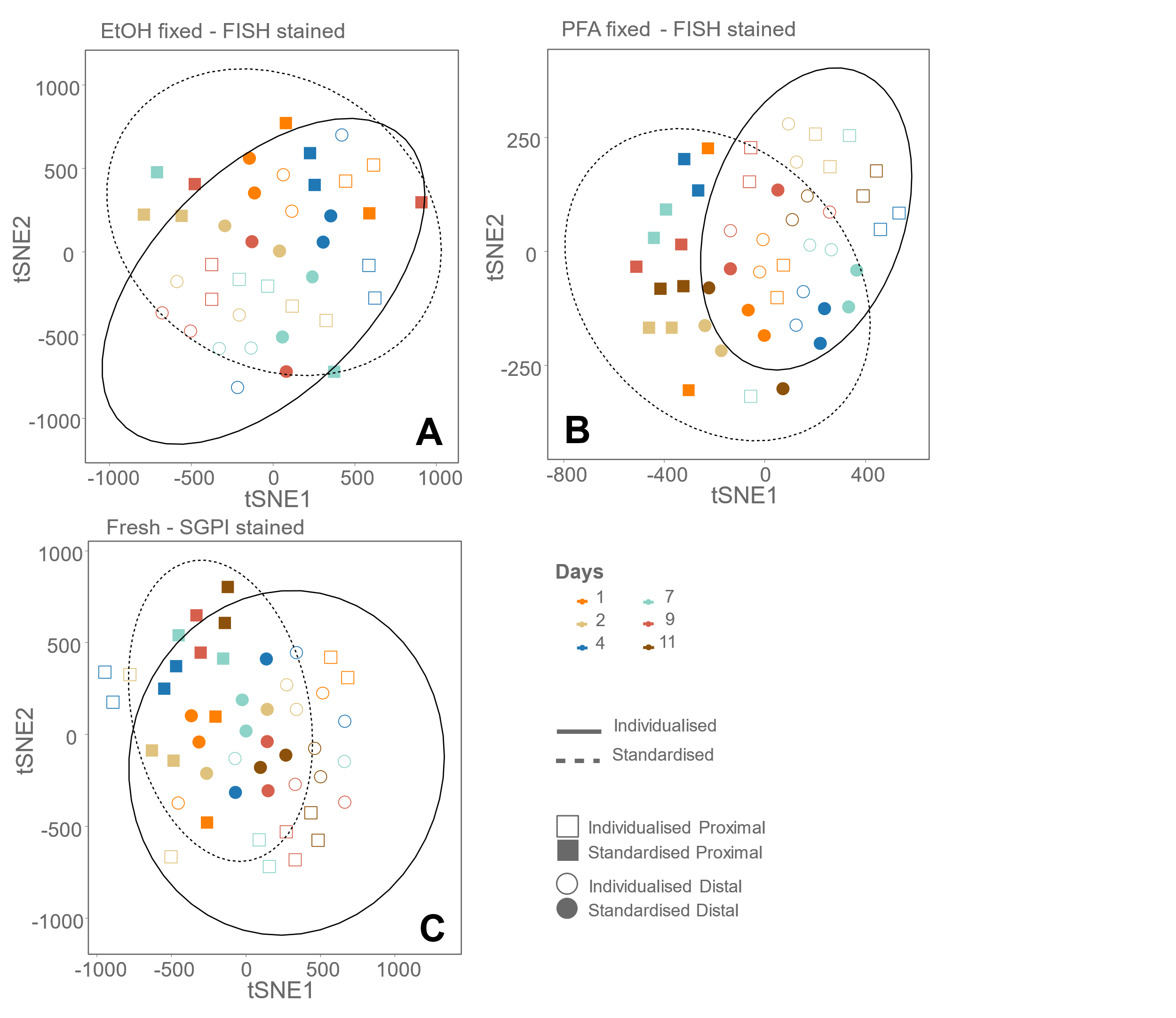


**Supplementary Figure 6** – Simulated gut experiment. tSNE dimensionality reduction based on Bray-Curtis dissimilarity matrix calculated based on (**A**) DAPI and FISH staining of EtOH -fixed cells (**B**) DAPI and FISH staining of PFA-fixed cells and (**C**) SGPI staining. The different reactors, based on individualised/standardised SHIME operation and distal/proximal colon vessel, are displayed by empty (○,□) and full (●, ■) circles and squares. Biological duplicates are shown. The ellipse is drawn on the 95% confidence level (based only on SHIME operation settings: individualised and standardised).


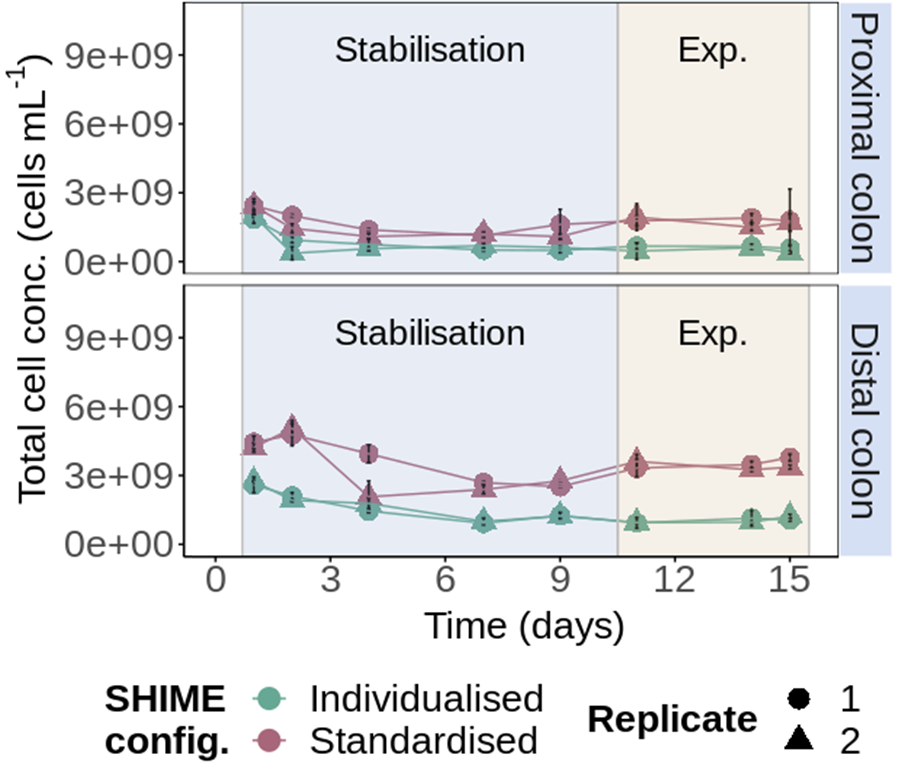


**Supplementary Figure 7** - The total cell concentrations (cells mL^−1^) between the standardised and individualised SHIMEs during the stabilisation and experimental phases (Exp., yellow background). Every SHIME replicates are designated with circles (replicate 1) or triangles (replicate 2).

**Supplementary Table 4** – The accuracy obtained by predictive modelling, five consecutive times, for the different staining methods in the different ecosystems.

| **Staining method** | **Average AUC** |
| --- | --- |
| Marine: Non-fixed SG | 0.9854 |
| Marine: Fixed SG | 0.9532 |
| Marine: Fixed DAPI | 0.8026 |
| **Marine: FISH** | **0.9408** |
| SHIME: Non-fixed SGPI | 0.9874 |
| SHIME: FISH EtOH | 0.9376 |
| **SHIME: FISH PFA** | **1** |
